# Editorial: Sleep and Psychological Trauma or Stress

**DOI:** 10.3389/fpsyt.2022.951217

**Published:** 2022-06-14

**Authors:** Seog Ju Kim

**Affiliations:** Department of Psychiatry, Sungkyunkwan University School of Medicine, Samsung Medical Center, Seoul, South Korea

**Keywords:** sleep, trauma, stress, posttraumatic stress disorder, depression

Restorative sleep plays a critical role in maintaining physical and mental health. Sleep disturbances frequently co-occur with trauma or stress related disorder such as posttraumatic stress disorder (PTSD). It has long been thought that interventions focusing on trauma itself would eventually reduce disturbed sleep. However, accumulating evidence have shown that sleep disturbances play a central role in both the development and maintenance of PTSD and therefore require clinical attention. As well as traumatic stress, stressful experiences are also associated with sleep (1). Stress can worsen the sleep quality, while poor sleep can also interfere with emotional processing of stressful experiences. The current Research Topic represents a collection of papers investigating the relationship between sleep abnormalities and psychological trauma or stress, as well as studies analyzing potential mechanisms connecting these pathologies.

In the current Research Topic, 1 review article and 1 original article on the association between PTSD and sleep were published. The review article by Lancel et al. introduced sleep disorders which have been suggested as predisposing, facilitating, and perpetuating factors of PTSD. This review also introduces non-pharmacological and pharmacological treatments for the four major sleep disorders commonly accompanying PTSD. Authors strongly recommended an initial comprehensive evaluation and timely treatment for comorbid sleep disorders for PTSD patients.

In the article by Denis et al., the relationship between sleep microarchitecture and symptom severity in trauma-exposed participants with and without PTSD were investigated. Contrary to previous studies, beta power was increased in trauma-exposed controls (TEC) compared to PTSD. Spectral power in the beta frequency was associated with reduced symptoms. Their results suggest an adaptive role of beta power during sleep for individuals exposed to trauma.

There are many stressful situations in hospital both for patients and health care workers, and these stressful situations can be associated with sleep. The current Research Topic covers the relationship between sleep and hospital-related stressful situations such as COVID-19, HIV infection, operation, and pregnancy. Cleper et al. reported that the influence of COVID-19 work-related stressors on sleep in physicians and nurses working in designated COVID-19 wards. COVID-19 frontline health care workers were more likely to report sleep difficulties compared to non-COVID-19 health care workers in the same hospital. In particular, the negative experiences, mainly witnessing patient's physical suffering or death, partially explained the association between COVID-19 work and sleep disturbances.

The reliability and validity of the Chinese version of Pittsburgh Sleep Quality Index (PSQI) in people living with HIV were assessed by Yan et al.. PSQI was used to measure sleep quality 5 years after HIV diagnosis. Sleep disturbances were associated with less income, higher CD4 counts, antiretroviral treatment initiation, exercise, depression, and higher stress levels. Authors reported that the Chinese version of PSQI is feasible for use among people living with HIV considering its internal consistency and the construct validity.

Applicability of intranasal dexmedetomidine (DEX) in the treatment of preoperative insomnia was reported by Zeng et al.. Patients with preoperative insomnia were divided into two groups of intranasal DEX and normal saline (NS). Intranasal DEX shorten sleep latency and lengthen total sleep time compared to intranasal NS. The intranasal DEX application also reduced insomnia and drowsiness. They suggest that intranasal DEX can improve preoperative insomnia, especially for patients who are reluctant to take sleeping pills before surgery.

Most women complained sleep disturbances during perinatal/postnatal period, which is associated with physiological and psychological change during pregnancy (2). Two papers on the association between sleep and perinatal/postnatal depression were reported by Bao et al.. Poor sleep trajectories during the perinatal period were related to postpartum depression. Authors suggest that screening for prenatal sleep problems would be helpful for identifying the onset of perinatal depression. Bao et al. also reported that patients with prenatal depression showed poor sleep quality and decision-making function especially when they had suicidal ideation. This study suggests that sleep disturbance and impaired decision-making function may be risk factors for suicidal ideation in prenatal depression.

The current Research Topic also covers the association between sleep and common psychological stress of young adults. Manzar et al. demonstrates that the interrelationship between psychological stress, poor sleep, inadequate sleep hygiene, and anxiety in university students. They suggest a need to address the various aspects of mental health and its diverse sleep correlates in university students.

Barbeau et al. investigated the impact of recent troubling experiences on dream characteristics. Individuals who experienced a recent troubling event reported a higher frequency of nightmares and more emotionally negative dream. Nightmare and oneiric dream were more common in young adults. Authors suggest that dysphoric dreams might serve as potential proxies of mental health status and developmental stages.

Taken together, this Research Topic covered several diverse of sleep disturbance and psychological trauma or stress, and can update readers with the latest research in the field of sleep medicine, psychosomatic medicine and psychiatry. These articles will provide insights to the clinicians and researchers, and help them to further explore the relationship between sleep and psychological trauma or stress with the goal of developing effective therapeutic strategies.

## Author Contributions

The author confirms being the sole contributor of this work and has approved it for publication.

## Funding

This research was supported by the National Research Foundation of Korea (NRF) grant funded by the Korean government (MSIT) (No. 2022R1A2C2008417), the Bio and Medical Technology Development Program of the National Research Foundation (NRF) funded by the Korean government (MSIT) (No. 2020M3E5D9080561), and a grant of the Korea Health Technology R&D Project through the Korea Health Industry Development Institute (KHIDI) funded by the Ministry of Health & Welfare, Republic of Korea (No. HR21C0885).

## Conflict of Interest

The author declares that the research was conducted in the absence of any commercial or financial relationships that could be construed as a potential conflict of interest.

## Publisher's Note

All claims expressed in this article are solely those of the authors and do not necessarily represent those of their affiliated organizations, or those of the publisher, the editors and the reviewers. Any product that may be evaluated in this article, or claim that may be made by its manufacturer, is not guaranteed or endorsed by the publisher.
